# Identifying gram-positive cocci in dermatoscopes and smartphone adapters using MALDI-TOF MS: a cross-sectional study^☆^^☆☆^

**DOI:** 10.1016/j.abd.2019.11.004

**Published:** 2020-03-21

**Authors:** Maurício de Quadros, Roberto Carlos Freitas Bugs, Renata de Oliveira Soares, Adriana Medianeira Rossato, Lisiane da Luz Rocha, Pedro Alves d’Azevedo

**Affiliations:** a Gram-positive Cocci Laboratory, Universidade Federal de Ciências da Saúde de Porto Alegre, Porto Alegre, RS, Brazil; b Department of Dermatology, Hospital Santa Casa de Misericórdia de Porto Alegre, Porto Alegre, RS, Brazil

**Keywords:** Dermoscopy, Gram-positive cocci, Mass spectrometry, Microbial sensitivity tests

## Abstract

**Background:**

The increasingly frequent use of dermoscopy makes us think about the possibility of transfer of microorganisms, through the dermatoscope, between doctor and patients.

**Objectives:**

To identify the most frequent gram-positive cocci in dermatoscopes and smartphone adapters, as well as the resistance profile, and to evaluate the factors associated with a higher risk of bacterial contamination of the dermatoscopes.

**Methods:**

A cross-sectional study was carried out with 118 dermatologists from Porto Alegre/Brazil between September 2017 and July 2018. Gram-positive cocci were identified by MALDI-TOF MS and habits of use of the dermatoscope were evaluated through an anonymous questionnaire.

**Results:**

Of the dermatoscopes analysed, 46.6% had growth of gram-positive cocci on the lens and 37.3% on the on/off button. The microorganisms most frequently found were *S. epidermidis*, *S. hominis* and *S. warneri*. Attending a hospital, using the dermatoscope at the hospital, with inpatients and in the intensive care unit were significantly associated with colonisation by gram-positive cocci. The highest resistance rates were observed for penicillin, erythromycin and sulfamethoxazole-trimethoprim.

**Study limitations:**

The non-search of gram-negative bacilli, fungi and viruses. Moreover, the small number of adapters did not make it possible to better define if the frequency differences were statistically significant.

**Conclusion:**

Coagulase-negative staphylococci were frequently identified. *S. aureus* was detected only on the lens.

## Introduction

Dermoscopy is an excellent diagnostic tool in the daily practice of the dermatologist. In recent years, smartphone adapters have been used to photograph skin lesions, such as melanocytic nevi, and allow their follow-up, as well as facilitate case discussions among dermatologists.

These technological innovations have enabled health professionals to quickly access a wider range of information: using smartphones and tablets it is easier to search for articles, rapidly access relevant topics in books and applications, discuss cases in groups with experts, and participate in teaching future health professionals.^1^, ^2^ However, the indiscriminate use of such objects provides a new challenge: the possible transference of microorganisms, with or without pathogenic potential, from these devices to the hands of professionals, or vice versa, or person to person transference. For example, patients with nasal colonization by *Staphylococcus aureus* are 2–9 times more likely to have *S. aureus* infection.^3^ The most frequently identified pathogens associated with healthcare-associated infections were Coagulase-Negative Staphylococci (CoNS) (15%), *S. aureus* (15%), *Enterococcus* sp. (12%), *Candida* sp. (11%), followed by several gram-negative bacilli.^4^ These healthcare-related infections represent a major challenge for the health system and are associated with significant costs, morbidity, and mortality. At any one time, up to 7% of patients in developed and 10% in developing countries will acquire at least one health care-associated infection.^5^

Few studies have evaluated the contamination of microorganisms in dermatoscopes and there are no studies on smartphone adapters. A study in Switzerland analysed the bacterial presence on the lenses of dermatoscopes belonging to 10 dermatologists (*n* = 10) involved in the care of patients from the dermatology outpatient clinics of two Swiss hospitals. Of the 112 swabs taken, 65% showed the growth of non-pathogenic bacteria (including all the CoNS, Streptococcus α and γ-haemolytic, Corynebacterium, Bacillus and Lactobacillus). Methicillin-Susceptible Staphylococcus Aureus (MSSA) was found on three occasions (on the three swabs, for dermoscopy, immersion oil had been used on the apparatus rather than isopropyl alcohol).^6^ In Austria, researchers studied the spectrum of microorganisms in 4 dermatoscopes (lens and body) used in the department of dermatology at a Vienna hospital after dermoscopy in 39 patients and found *S. epidermidis* in 74% of the devices and *S. aureus* in 7%. In the United Kingdom, Chattopadhyay et al. (2014) evaluated bacterial growth on 9 dermatoscope lenses on 60 occasions (30 before dermoscopy and 30 after). An alcohol-gel containing 70% ethanol was used as the immersion liquid. The authors found Methicillin-Resistant *S. Aureus* (MRSA) in 10% of the experiments (all from swabs obtained after dermoscopy).^7^

The present researchers have sought to identify the gram-positive cocci most commonly found on dermatoscopes and smartphone adapters and to assess the factors associated with the increased risk of bacterial contamination.

## Methods

Between September 2017 and July 2018, we conducted a cross-sectional study among dermatologists who attended at a dermatology outpatient clinic of a public hospital and in private practices. The doctors answered an anonymous questionnaire containing demographic information and about the habits of use of the dermatoscope, and provided their devices for bacteriological analysis via the swab technique. Physicians who did not wish to fill out the questionnaire or provide their dermatoscopes or cell phone adapters for swabbing were excluded.

Samples were collected from two or three previously defined sites on the dermatoscopes: the lens, the on/off button and the outside of the smartphone adapter (for professionals who used that device). The swabs were sealed, labelled and forwarded for analysis. In the laboratory, the swabs were placed in tubes containing BHI enriched medium (brain-heart infusion) (Sigma Aldrich, Merck, Germany) and left in the oven at 35 ±2 °C for 24 h. In the presence of turbidity (in which case the test is considered positive, i.e. there was bacterial growth), the broths were seeded in 5% sheep-blood agar plates (Biomeriéux, Marcy L’Etoile, France) using a sterile loop. The plates were then returned to the oven at 35 ±2 °C for 24 h. The plates showing bacterial growth were placed in skimmed milk and frozen for later identification. Samples that clouded in the BHI broth were subsequently thawed and, again, seeded on blood agar plates for identification by MALDI-TOF MS. In this study, we used the Bruker Daltonics platform (microflex LT; Bruker Daltonik GmbH, Bremen, Germany).

To test for susceptibility, after 24 h in culture with sheep-blood agar, the colonies were incubated at 35 ±2 °C for 18–24 h and tested for the following antibiotics: penicillin (10 U), cefoxitin (30 μg), erythromycin (15 μg), clindamycin (2 μg), levofloxacin (5 μg), sulfamethoxazole–trimethoprim (1.25–23.75 μg), linezolid (30 μg), tetracycline (30 μg), gentamicin (10 μg) and rifampicin (5 μg). The plates were analysed according to the recommendations of the Clinical and Laboratory Standards Institute (CLSI, 2018).^8^ According to the diameter of the inhibition halo, the samples were classified as sensitive, intermediate resistance or resistant. *S. aureus* ATCC 25923 was used to control the quality of the antibiotic discs, according to standard disc-diffusion test procedures.

The research was approved by the Research Ethics Committee of the Santa Casa de Misericórdia de Porto Alegre (protocol n° 9396017.5.0000.5335), Universidade Federal de Ciências da Saúde (protocol n° 69396017.5.0001.5345) and Secretaria Estadual de Saúde do Rio Grande do Sul (protocol n° 9396017.5.3002.5312). All the participants included in the study signed an Informed Consent Term.

The data was entered into the Excel program and then exported to the SPSS v. 20.0 for statistical analysis. Qualitative variables were described by frequency and percentages. Symmetrically distributed quantitative variables were described using the mean and standard deviation, while for those with an asymmetric distribution; the median and the interquartile range were used. Categorical variables were compared using the Chi-square test or Fisher's exact test. The quantitative variables were compared using Mann-Whitney test or Student *t*-Test. A significance level of 5% was considered for the established comparisons.

Regarding the sample size, with approximately 59 dermatologists per group, we were able to detect a difference of 20 percentage points in the frequency of bacterial colonization. We considered a baseline colonization value of 5% (in the cited literature, the value ranges from 2.7% to 10%), a power of 80% and a significance of 5%.

## Results

**Figure 1:**
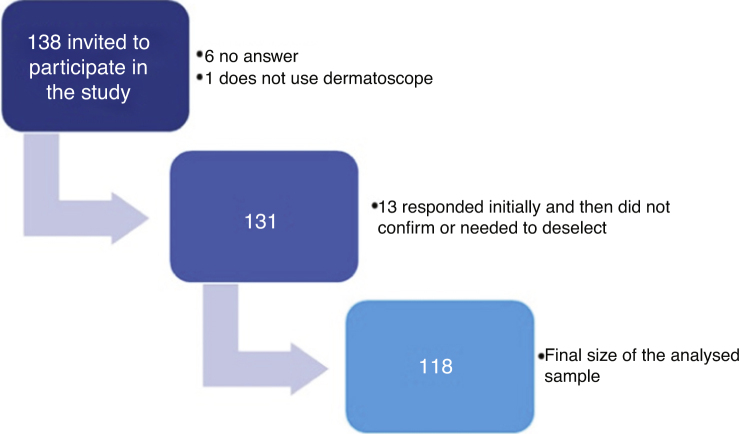
Sample of dermatologist's flowchart.

A total of 138 dermatologists were invited to participate in the study (Fig. 1). The characteristics of the 118 dermatologists whose devices were analysed are described in table 1.

**Table 1:** Characteristics of the dermatologists in the sample and the use of the dermatoscope.

Variable	Descriptive measures
*Age, years – mean* *±* *SD*	36.4 ± 8.4

*Sex, n (%)*	
Male	16 (13.5)
Female	102 (86.5)
Time as a dermatologist, median – years (interquartile range)	6.5 (2*–*15)

*Where do you attend patients?* – *n (%)*	
In the private office	47 (39.8)
At hospital	25 (21.2)
In the private office and at hospital	46 (39)
Number of times the dermatoscope id used per day – median (interquartile range)	15 (10*–*20)
Amount of time the dermatoscope is used during consultation, in minutes – median (interquartile range)	5 (3*–*7)

*Model of dermatoscope, n (%)*	
DL100	6 (5.1)
DL200	2 (1.7)
DL3	24 (20.3)
DL4	34 (28.8)
Hybrid	42 (35.6)
MiniHeine or Heine	8 (6.8)
Wellch Allyn	1 (0.8)
Veos Canfield	1 (0.8)

*Does the device touch the patient's skin during the examination?* – *n (%)*	
Yes	92 (78)
No	26 (22)

*Where do you keep the dermatoscope? – n (%)*	
Coat pocket	68 (57.6)
Desktop	76 (64.4)
Wardrobe	21 (17.8)
Case	15 (12.7)

*Do you use the dermatoscope in the private office? – n (%)*	
Yes	102 (86.4)
No	16 (13.6)

*Do you use the dermatoscope at the hospital? – n (%)*	
Yes	64 (54.2)
No	54 (45.8)

*Do you use the dermatoscope with inpatients? – n (%)*	
Yes	39 (33)
No	79 (67)

*Do you use the dermatoscope in the ICU? – n (%)*	
Yes	20 (17)
No	98 (83)

*Have you attended an inpatient in isolation due to multidrug resistant bacteria in the last 30 days? – n (%)*	
Yes	15 (12.7)
No	103 (87.3)

*Have you used the dermatoscope on an inpatient with multidrug resistant bacteria in the last 30 days? – n (%)*	
Yes	2 (1.7)
No	116 (98.3)

*Do you use any cleanser for the dermatoscope? – n (%)*	
Yes	92 (78)
No	26 (22)

*Do you use a smartphone adapter? – n (%)*	
Yes	27 (22.9)
No	91 (77.1)

SD, standard deviation; ICU, intensive care unit.

Of the dermatoscopes analysed, 46.6% had gram positive cocci colonies on the lens and 37.3% on the on/off button (Table 2).

**Table 2:** Frequency of bacterial colonization by gram-positive cocci in dermatoscopes and smartphone adapters.

Variable	*n*/total	Frequency (%)	95% CI
Bacterial growth in the dermatoscope (lens or on/off button)	70/118	59.3	49.9*–*68.3
Bacterial growth in the lens	55/118	46.6	37.4*–*56.0
Bacterial growth on the on/off button	44/118	37.3	28.6*–*46.7
Bacterial growth on smartphone adapter	10/27	37	19.4*–*57.6

CI, confidence interval.

The frequency of gram-positive cocci was higher among males, but the difference was not statistically significant (Table 3). The variables significantly associated with colonisation by gram-positive cocci were: being a resident. Attending a hospital or not attending exclusively in private office; Keeping the dermatoscope in the coat pocket; Using the dermatoscope at the hospital; With inpatients and; In the Intensive Care Unit (*p* < 0.05). Using a smartphone adapter was not associated with dermatoscope contamination.

**Table 3:** Categorical variables and their relation to the presence of bacterial contamination on the lens or on the on/off button.

Variable	Frequency (*n*)	Contamination by gram positive cocci (%)	*p*-Value
*Sex*			0.581^a^
Male	11	68.8	
Female	59	57.8	

*Resident*			0.013^a^
Yes	33	75	
No	37	50	

*Where do you attend?*			0.010^a^
Private office	20	42.6	
Hospital	18	72	
Private office and hospital	32	69.6	

*Where do you attend?*			0.005^a^
Only private office	20	42.6	
Only hospital or private office plus hospital	50	70.4	

*Model of dermatoscope*			0.361^a^
DL100 and DL200	4	50	
DL3, DL4, Veos Canfield	37	62.7	
Hybrid	26	61.9	
Wellch Allyn or Heine	3	33.3	

*Does it touch the skin?*			0.676^a^
Yes	56	60.9	
No	14	53.8	

*Keep the dermatoscope in the coat pocket*			0.019^a^
Yes	47	69.1	
No	23	46	

*Put the dermatoscope on the table*			0.819^a^
Yes	44	57.9	
No	26	61.9	

*Store the dermatoscope in a closet*			0.999^a^
Yes	12	57.1	
No	58	59.8	

*Store the dermatoscope in a case*			0.735^a^
Yes	10	66.7	
No	60	58.3	

*Do you use the dermatoscope in the private office?*			0.272^a^
Yes	58	56.9	
No	12	75.0	

*Do you use the dermatoscope at the hospital?*			0.001^a^
Yes	47	73.4	
No	23	42.6	

*Do you use the dermatoscope with inpatients?*			<0.001^a^
Yes	33	84.6	
No	37	46.8	

*Do you use the dermatoscope in the ICU?*			<0.001^a^
Yes	20	100	
No	50	51	

*Have you attended patients in isolation due to multidrug resistant bacteria in the last 30 days?*			0.143^a^
Yes	12	80	
No	58	56.3	

*Have you used the dermatoscope on a patient with multidrug resistant bacteria in the last 30 days?*			0.513^b^
Yes	2	100	
No	68	58.6	

*Do you clean the dermatoscope?*			0.348^a^
Yes	52	56.5	
No	18	69.2	

*Do you use a smartphone adapter?*			0.999^a^
Yes	16	59.3	
No	54	59.3	

ICU, intensive care unit.

a Chi-squared test.

b Fisher's exact test.

**Figure 2:**
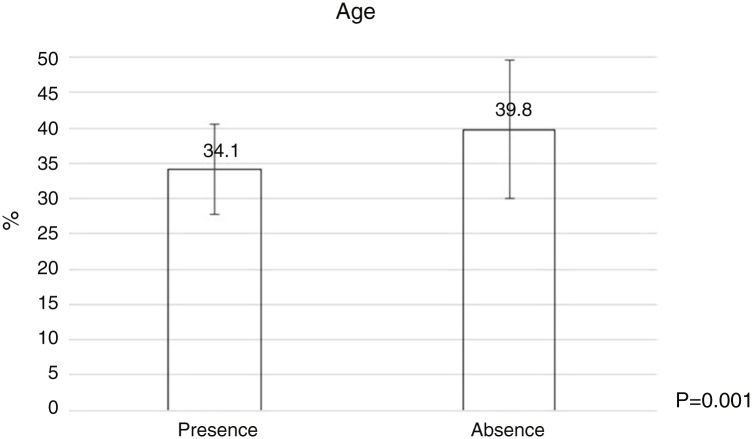
Age of dermatologists and their relation to the presence or absence of bacterial contamination on dermatoscopes. Statistic Test: Student *t*-test. Standard deviation in the group with gram-positive cocci 6.4. Standard deviation in the group without gram-positive cocci 9.8.

Bacterial contamination was more common among younger dermatologists (Fig. 2) and those wiht less time as dermatologist (Table 4), and a statistically significant relationship was found between the number of patients treated per day and the number of times they used the dermatoscope per day (Table 4).

**Table 4:** Numerical variables and their relation to the presence or absence of bacterial contamination on the lens or on the on/off button.

Variable	Presence of gram-positive median cocci (interquartile range)	Absence of gram-positive-median cocci (interquartile range)	*p*-Value
Time as a dermatologist, years	4 (1*–*11.25)	11 (4.25*–*18)	<0.001
Number of times the dermatoscope is used per day	15 (10*–*20)	10 (10*–*16.5)	0.004
Number of patients seen per day	20 (15*–*25)	15.5 (12.25*–*20)	0.035
Dermoscopy time per consultation, minutes	5 (3*–*7.25)	5 (3*–*5)	0.881
How many times a day do you access your mobile phone?	10 (4.75*–*15)	10 (5*–*15)	0.862

Statistical test used: Mann–Whitney test.

**Figure 3:**
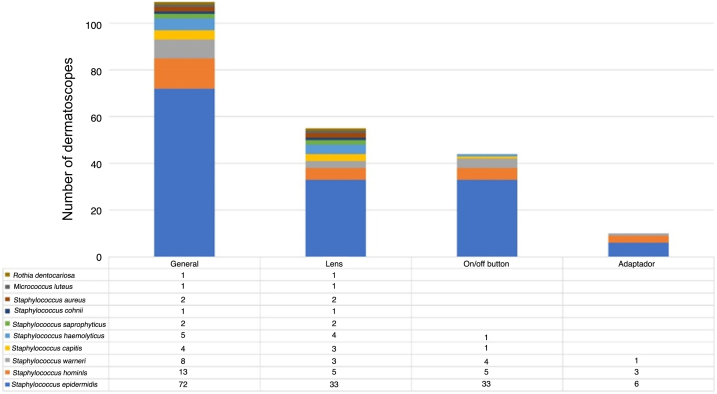
Gram-positive cocci identified by MALDI-TOF MS.

The microorganisms most frequently found were *S. epidermidis*, *S. hominis* and *S. warneri. S. aureus* was only detected on the lens (Fig. 3).

The highest resistance rates among the gram-positive cocci were found to be against penicillin, erythromycin, Sulfamethoxazole-Trimethoprim (SMT-TMP) and clindamycin (Table 5). Cefoxitin resistance was 6.6% and no microorganism was resistant to linezolid.

**Table 5:** Antimicrobial resistance profile of gram-positive cocci isolates obtained from dermatoscopes and smartphone adapters.

	Gram-positive cocci			
	Sensitivity		Resistance	
Antibiotic	*n*	%	*n*	%
Penicillin	26	23.6	84	77.4
Erythromycin	31	28.4	78	71.6
Clindamycin	75	68.8	34	31.2
Tetracycline	95	86.4	15	13.6
SMT-TMP	66	60.6	43	39.4
Cefoxitin	103	93.6	7	6.4
Gentamicin	104	94.5	6	5.5
Rifampicin	105	95.4	5	4.6
Levofloxacin	103	93.6	7	6.4
Linezolid	110	100	0	0

SMT-TMP, sulfamethoxazole-trimethoprim.

*S. epidermidis* presented a high rate of resistance to penicillin, erythromycin and SMP-TMP, whereas *S. hominis* presented greater resistance to erythromycin than penicillin. While *S. capitis* presented high resistance rates to several antibiotics, no case of resistance to clindamycin and gentamicin was found. The highest rates of resistance to penicillin were found for *S. warneri* and *S. haemolyticus*. All isolates of *S. haemolyticus* were resistant to penicillin and it had the highest frequencies of resistance to clindamycin, tetracycline, SMT-TMP and gentamicin (Table 6).

**Table 6:** Antimicrobial resistance profile of the isolates of the most frequent gram-positive cocci obtained from dermatoscopes and smartphone adapters.

	Frequency of resistance among gram-positive cocci (%)				
Antibiotic	*S. epidermidis*	*S. hominis*	*S. warneri*	*S. capitis*	*S. haemolyticus*
Penicillin	79.4	50	88.9	75	100
Erythromycin	73.6	83.3	55.6	50	80
Clindamycin	36.6	25	11.1	0	40
Tetracycline	15.1	8.3	0	25	40
SMT-TMP	44.4	25	22.2	50	60
Cefoxitin	6.8	0	0	25	20
Gentamicin	5.5	0	0	0	40
Rifampicin	4.1	0	0	25	0
Levofloxacin	5.5	0	0	75	0
Linezolid	0	0	0	0	0

SMT-TMP, sulfametoxazol-trimetropim.

## Discussion

The dermatoscopes were mainly colonized by bacteria from the cutaneous microbiota (CoNS), the most frequently found being *S. epidermidis*, which is accordance with the literature.^9^, ^10^ This microorganism has become the most common cause of primary bacteraemia and infection of medical devices, such as catheters, particularly in immunocompromised individuals and neonates. In contrast to *S. aureus*, which is much more virulent and synthesizes an array of toxins and other virulence factors, the main known virulence factor associated with *S. epidermidis* is its ability to form biofilms and colonize biomaterials. Again, in contrast to *S. aureus*, which is commonly located in the nasal mucosa, *S. epidermidis* can be easily transferred to the skin of other individuals through simple contact.^11^

Both *S. hominis*, the second most common microorganism found in our study, which is cited in the literature as one of the three CoNS most frequently found in neonatal blood cultures and immunosuppressed patients,^12^ and *S. warneri*, the third most common microorganism identified in dermatoscopes in our study, which some articles suggest is the second most frequent CoNS,^13^, ^14^ have the capacity to form biofilm,^15^, ^16^ and have been associated with bacteraemia, septicaemia, and endocarditis.^16^, ^17^

*S. capitis* rarely causes infection in adults, but a decreased susceptibility to vancomycin has been reported and a clonal population of methicillin-resistant *S. capitis* with vancomycin heteroresistance has spread among several neonatal ICUs in France and elsewhere.^18^ Ehlersson et al. evaluated *S. capitis* isolates from neonatal hemocultures in Sweden and found a 75% cefoxitin and gentamicin resistance rate, only 3% erythromycin resistance, and no case of resistance either to norfloxacin or SMT-TMP.^19^

We found that the dermatoscope can carry *S. aureus*. This bacteria colonizes the superficial layer of the skin, survives for a short period of time, and is often acquired by health professionals during direct contact with the patient (colonized or infected), environment, surfaces close to the patient, contaminated products and equipment.^20^, ^21^ In fact in our study, it was detected precisely on the lens but not on the on-off button or smartphone adapter, places more likely to be related to direct contact with the skin of the health professional.

Hands of health professionals may be persistently colonized by pathogenic microorganisms (such as *S. aureus*, gram-negative bacilli or yeasts), which in critical areas, such as intensive care units and units with immunocompromised or surgical patients, can play an important role as a cause of infection related to health care.^22^

In our study, cefoxitin resistance was considered low. Resistance to erythromycin was notably high in *S. hominis* isolates (83.3%), a fact already mentioned in other studies.^23^ Szczuka et al. (2016) found an erythromycin resistance rate of 75% in isolates from blood and surgical wounds of hospitalized patients.^24^ The highest rates of resistance to various antibiotics were seen in *S. haemolyticus*, as previously reported.^23^ Recent studies have cited *S. haemolyticus* as the second CoNS, after *S. epidermidis*, most frequently isolated from clinical cases, including sepsis patients.^25^, ^26^, ^27^

This is the first study in the literature to evaluate antimicrobial resistance of CoNS in dermatoscopes and smartphone adapters. Knowing the resistance pattern of CoNS in dermatoscopes, and on our own skin, is important given that this bacterial group can act as reservoir of antimicrobial resistant genes by horizontal transfer between staphylococcal species. Furthermore, they may be acquired by *S. aureus*,^26^, ^28^, ^29^ and subsequently transferred between dermatologists and their patients, especially physicians working in hospital settings where antimicrobial resistance rates are highest.^26^ According to a cohort of 2518 patients in Israel, identifying the CoNS resistance patterns obtained by blood cultures, even when contaminants, could help predict mortality and correct empirical antibiotic therapy.^30^

Among the limitations of our study is the non-screening of gram-negative bacteria, fungi, and viruses. Moreover, the small number of adapters meant it we were unable better determine whether the differences in frequency were statistically significant.

## Conclusions

We identified a high frequency of gram-positive cocci on the tested devices. *Staphylococcus epidermidis* was the most frequently observed, both on the lens, the on/off button and the smartphone adapter. *S. aureus* was detected only on the lens.

This study concerns the association between the dermatologist and the contamination of dermatoscopes. Professionals should take measures to prevent contamination of their devices and cross-colonization with their patients.

## Financial support

Coordenação de Aperfeiçoamento de Pessoal de Nível Superior (CAPES), Conselho Nacional de Pesquisa e Desenvolvimento Tecnológico (CNPq) and Fundação de Amparo à Pesquisa do Rio Grande do Sul (FAPERGS).

## Authors’ contributions

Maurício de Quadros: Statistic analysis; approval of the final version of the manuscript; conception and planning of the study; elaboration and writing of the manuscript; obtaining, analysis, and interpretation of the data; effective participation in research orientation; critical review of the literature; critical review of the manuscript.

Roberto Carlos Freitas Bugs: Approval of the final version of the manuscript; conception and planning of the study; obtaining, analysis, and interpretation of the data; critical review of the literature.

Renata de Oliveira Soares: Approval of the final version of the manuscript; conception and planning of the study; obtaining, analysis, and interpretation of the data; effective participation in research orientation.

Adriana Medianeira Rossato: Approval of the final version of the manuscript; conception and planning of the study; elaboration and writing of the manuscript; obtaining, analysis, and interpretation of the data; effective participation in research orientation; critical review of the literature; critical review of the manuscript.

Lisiane da Luz Rocha: Approval of the final version of the manuscript; conception and planning of the study; elaboration and writing of the manuscript; obtaining, analysis, and interpretation of the data; critical review of the manuscript.

Pedro Alves d’Azevedo: Statistic analysis; approval of the final version of the manuscript; conception and planning of the study; elaboration and writing of the manuscript; obtaining, analysis, and interpretation of the data; effective participation in research orientation; critical review of the literature; critical review of the manuscript.

## Conflicts of interest

None declared.

## References

[1] Manning M.L., Davis J., Sparnon E., Ballard R.M. (2013). iPads, droids, and bugs: infection prevention for mobile handheld devices at the point of care. Am J Infect Control.

[2] Visvanathan A., Gibb A.P., Brady R.R. (2011). Increasing clinical presence of mobile communication technology: avoiding the pitfalls. Telemed J E Health.

[3] Wenzel R.P., Perl T.M. (1995). The significance of nasal carriage of *Staphylococcus aureus* and the incidence of postoperative wound infection. J Hosp Infect.

[4] Hidron A.I., Edwards J.R., Patel J., Horan T.C., Sievert D.M., Pollock D.A. (2008). NHSN annual update: antimicrobial-resistant pathogens associated with healthcare-associated infections: annual summary of data reported to the national healthcare safety network at the centers for disease control and prevention, 2006–2007. Infect Control Hosp Epidemiol.

[5] World Health Organization [Internet]. Geneva; c2016. Guidelines on core components of infection prevention and control programmes at the national and acute health care facility level. Availabre from: http://www.who.int/infection-prevention/publications/ipc-components-guidelines/en/ [cited 30.06.18]. 27977095

[6] Häusermann P., Widmer A., Itin P. (2006). Dermatoscope as vector for transmissible diseases – no apparent risk of nosocomial infections in outpatients. Dermatology.

[7] Chattopadhyay M., Blackman Northwood M., Ward B., Sule J., Burrows N.P. (2014). Are dermatoscopes a potential source of nosocomial infection in dermatology clinics?. Clin Exp Dermatol.

[8] CLSI. Performance Standards for Antimicrobial Susceptibility Testing. 28th ed. CLSI supplement M100. Wayne, PA: Clinical and Laboratory Standards Institute, 2018.

[9] Stauffer F., Kittler H., Forstinger C., Binder M. (2001). The dermatoscope: a potential source of nosocomial infection?. Melanoma Res.

[10] Cavanagh J.P., Wolden R., Heise P., Esaiassen E., Klingenberg C., Aarag Fredheim E.G. (2016). Antimicrobial susceptibility and body site distribution of community isolates of coagulase-negative staphylococci. APMIS.

[11] Fey P.D., Olson M.E. (2010). Current concepts in biofilm formation of Staphylococcus epidermidis. Future Microbiol.

[12] Al Wohoush I., Rivera J., Cairo J., Hachem R., Raad I. (2011). Comparing clinical and microbiological methods for the diagnosis of true bacteraemia among patients with multiple blood cultures positive for coagulase-negative staphylococci. Clin Microbiol Infect.

[13] Mehr S.S., Sadowsky J.L., Doyle L.W., Carr J. (2002). Sepsis in neonatal intensive care in the late 1990s. J Paediatr Child Health.

[14] Cimiotti J.P., Haas J.P., Della-Latta P., Wu F., Saiman L., Larson E.L. (2007). Prevalence and clinical relevance of *Staphylococcus warneri* in the neonatal intensive care unit. Infect Control Hosp Epidemiol.

[15] Mendoza-Olazarán S., Morfin-Otero R., Rodríguez-Noriega E., Llaca-Díaz J., Flores-Treviño S., González-González G.M. (2013). Microbiological and molecular characterization of *Staphylococcus hominis* isolates from blood. PLoS One.

[16] Szczuka E., Krzyminska S., Kaznowski A. (2016). Clonality, virulence and the occurrence of genes encoding antibiotic resistance among *Staphylococcus warneri* isolates from bloodstream infections. J Med Microbiol.

[17] d’Azevedo P.A., Trancesi R., Sales T., Monteiro J., Gales A.C., Pignatari A.C. (2008). Outbreak of *Staphylococcus hominis* subsp. novobiosepticus bloodstream infections in Sao Paulo city, Brazil. J Med Microbiol.

[18] Rasigade J.P., Raulin O., Picaud J.C., Tellini C., Bes M., Grando J. (2012). Methicillin-resistant *Staphylococcus capitis* with reduced vancomycin susceptibility causes late-onset sepsis in intensive care neonates. PLoS One.

[19] Ehlersson G., Hellmark B., Svartström O., Stenmark B., Söderquist B. (2017). Phenotypic characterization of coagulase-negative staphylococci isolated from blood cultures in newborn infants, with a special focus on *Staphylococcus capitis*. Acta Paediatr.

[20] Kramer A., Schwebke I., Kampf G. (2006). How long do nosocomial pathogens persist on inanimate surfaces? A systematic review. BMC Infect Dis.

[21] Kapil R., Bhavsar H.K., Madan M. (2015). Hand hygiene in reducing transient flora on the hands of healthcare workers: an educational intervention. Indian J Med Microbiol.

[22] Rotter M.L., Russell, Hugo, Ayliffe's (2004). Special problems in hospital antisepsis. Principles and practice of disinfection, preservation and sterilization.

[23] De Vecchi E., George D.A., Romano C.L., Pregliasco F.E., Mattina R., Drago L. (2018). Antibiotic sensitivities of coagulase-negative staphylococci and *Staphylococcus aureus* in hip and knee periprosthetic joint infections: does this differ if patients meet the International Consensus Meeting Criteria?. Infect Drug Resist.

[24] Szczuka E., Makowska N., Bosacka K., Stotwinska A., Kaznowski A. (2016). Molecular basis of resistance to macrolides, lincosamides and streptogramins in *Staphylococcus hominis* strains isolated from clinical specimens. Folia Microbiol (Praha).

[25] Silva P.V., Cruz R.S., Keim L.S., Paula G.R., Carvalho B.T., Coelho L.R. (2013). The antimicrobial susceptibility, biofilm formation and genotypic profiles of *Staphylococcus haemolyticus* from bloodstream infections. Mem Inst Oswaldo Cruz.

[26] Becker K., Heilmann C., Peters G. (2014). Coagulase-negative staphylococci. Clin Microbiol Rev.

[27] Czekaj T., Ciszewski M., Szewczyk E.M. (2015). Staphylococcus haemolyticus – an emerging threat in the twilight of the antibiotics age. Microbiology.

[28] Courvalin P. (1994). Transfer of antibiotic resistance genes between gram-positive and gram-negative bacteria. Antimicrob Agents Chemother.

[29] Ochman H., Lawrence J.G., Groisman E.A. (2000). Lateral gene transfer and the nature of bacterial innovation. Nature.

[30] Obolski U., Alon D., Hadany L., Stein G.Y. (2014). Resistance profiles of coagulase-negative staphylococci contaminating blood cultures predict pathogen resistance and patient mortality. J Antimicrob Chemother.

